# Giant renal angiomyolipoma: A case report

**DOI:** 10.1016/j.eucr.2021.101736

**Published:** 2021-05-30

**Authors:** Maher Al-Hajjaj

**Affiliations:** Department of Urology, Aleppo University Hospital, Aleppo, Syria

**Keywords:** Angiomyolipoma, Case report, Nephrectomy

## Abstract

Renal angiomyolipoma (AML) is a rare type of renal tumor. It consists of blood vessels, smooth muscle components, and mature adipose tissue. We report a rare case of giant renal angiomyolipoma in 36 years old male who had two months of intermittent mild abdominal pain. Computed tomography showed a right renal mass with 35 cm in diameter.

The mass was removed by surgery successfully. The patient had a good health course after surgery.

## Introduction

Angiomyolipoma (AML) is the most common benign renal tumor diagnosed in clinical practice. It is composed of a mix of dysmorphic blood vessels, smooth muscle components, and mature adipose tissue.^1^

AML can occur sporadically or may be associated with tuberous sclerosis complex (TSC) or sporadic lung lymphangioleiomyomatosis (LAM).^2^

The incidence rate of AML is about 0.3 %–3.0%. Tumors more than 10 cm (referred to as “giant” AMLs) are rare. Only a few reported cases, had giant tumors larger than 20 cm.^3^

Herein, we reported a very rare case of giant renal angiomyolipoma removed successfully by radical right nephrectomy.

## Case presentation

**Fig. 1:**
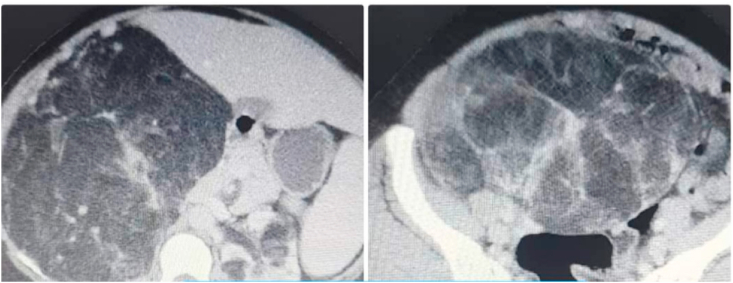
Computed tomography shows right renal giant mass.

A 36-year-old male patient presented with ambiguous abdominal pain, generalized fatigue, one episode of gross hematuria, and low back pain. Past medical history is remarkable for diabetes mellitus type 2 in his father. His symptoms started two months ago which treated by unknown drugs prescribed by his general practitioner. On examination, he was slightly pale, with mild abdominal tenderness with large swelling solid in consistency. Laboratory test findings showed hemoglobin of 9.6 g/dl. Other blood tests were in a normal range. An abdominal computed tomography revealed a right giant renal mass with a diameter of 35 cm. The chest x-ray was normal (Fig. 1).

Because of a large abdominal mass with two months of symptoms, we decided to do surgery.

**Fig. 2:**
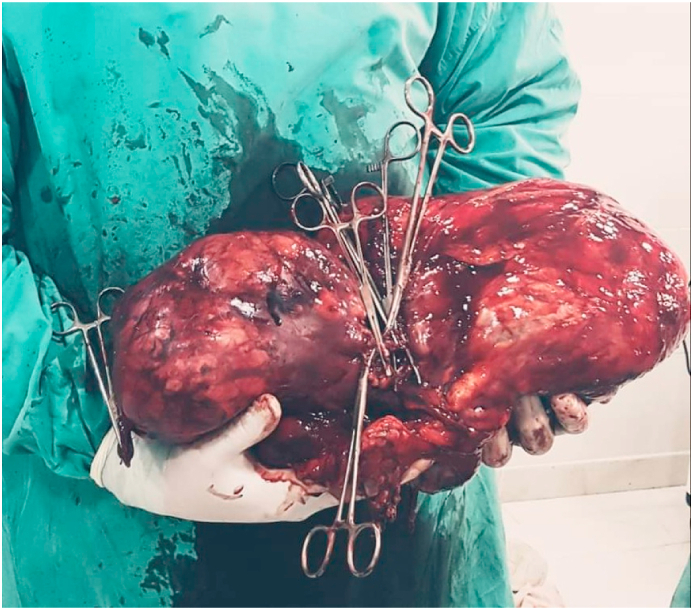
The whole mass after resection.

After taking the patient consent, he underwent a right radical nephrectomy in an open transabdominal approach. The patient had 1.5 L blood loss intraoperative. He was transferred with two units of red blood cells. The total weight was 7000 gr (Fig. 2).

Postoperation, he was in good health with no complaints. We removed the peritoneal drainage on the 4th day. After 7 days of surgery, he was discharged.

**Fig. 3:**
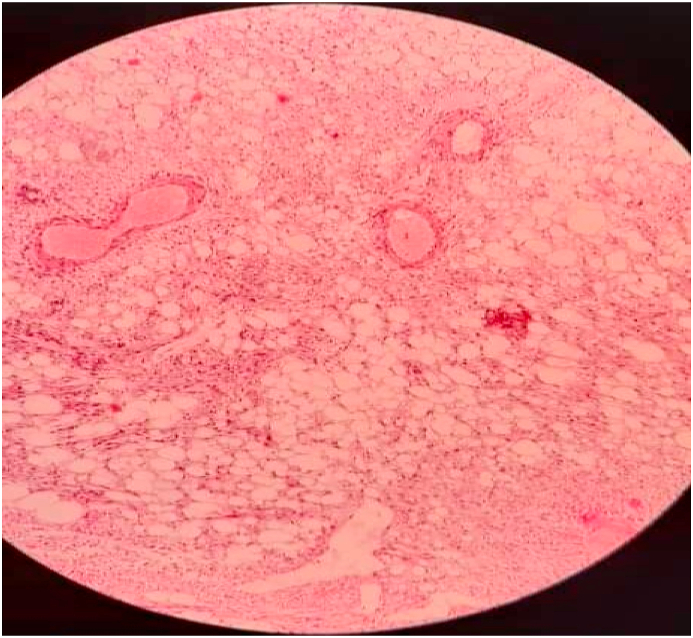
Microscopic view of classic angiomyolipoma.

The pathologist reported typical renal angiomyolipoma. Microscopic examination of serial sections revealed a neoplastic proliferation composed of mature adipose tissue, tortuous thick-walled blood vessels lacking elastic tissue lamina, and bundles of smooth muscle that seem to emanate from the vessel walls (Fig. 3). All surgical margins were free.

## Discussion

Renal angiomyolipoma is uncommon benign tumor; for the general population, this incidence stand between 0.07% and 0.3% with most commonly manifesting in middle aged woman.^4^

Most AML are benign and asymptomatic unless the tumor size reaches 4 cm or more.^5^

Clinical manifestations of AML include abdominal pain, distention and hematuria, although small masses are often asymptomatic.^1^

Surgical treatment is not recommended as a first-line therapy for AML. The indications for surgical treatment include suspicion of malignancy, symptoms, and a risk of hemorrhage.^2^

Mortality and morbidity of AML is associated with the risk of hemorrhage and the invasion of the lesion to adjacent normal renal parenchyma leading to chronic kidney disease and even end-stage renal disease.^5^

Our patient had two months of abdominal pain and a sensation of fullness. Next, he had low back pain, fatigue, and gross hematuria. These symptoms led him to ask for more evaluation in our center.

Abdominal examination showed a mass of solid consistency. That raised the suspicion of malignancy. Computed tomography demonstrated a right giant renal mass 35 cm in diameter that extends to the pelvis. Left renal was normal.

After two days, we agreed to do surgery because of the huge mass.

By transabdominal approach, we removed the giant mass. We had some bleeding, but we transferred two units of red blood cells. Peritoneal drainage was placed.

After, seven days in the ward, the patient was recovered well and was discharged.

Histopathology showed classic type renal angiomyolipoma.

## Conclusion

Giant renal angiomyolipoma is a rare entity. Few cases reported sporadic renal angiomyoplipoma greater than 20 cm. Our patient is considered a challenging case in daily urological practice.
